# Analysis of genetic diversity and population structure among cultivated potato clones from Korea and global breeding programs

**DOI:** 10.1038/s41598-022-12874-2

**Published:** 2022-06-21

**Authors:** Kwang Ryong Jo, Seungho Cho, Ji-Hong Cho, Hyun-Jin Park, Jang-Gyu Choi, Young-Eun Park, Kwang-Soo Cho

**Affiliations:** 1 Highland Agriculture Research Institute, National Institute of Crop Science, Rural Development Administration, Pyeongchang, 25342 Republic of Korea; 2 Department of Central Area Crop Science, National Institute of Crop Science, Rural Development Administration, Suwon, 16429 Republic of Korea; 3 Department of Southern Area Crop Science, National Institute of Crop Science, Rural Development Administration, Miryang, 50424 Republic of Korea

**Keywords:** Genetics, Agricultural genetics

## Abstract

Characterizing the genetic diversity and population structure of breeding materials is essential for breeding to improve crop plants. The potato is an important non-cereal food crop worldwide, but breeding potatoes remains challenging owing to their auto-tetraploidy and highly heterozygous genome. We evaluated the genetic structure of a 110-line Korean potato germplasm using the SolCAP 8303 single nucleotide polymorphism (SNP) Infinium array and compared it with potato clones from other countries to understand the genetic landscape of cultivated potatoes. Following the tetraploid model, we conducted population structure analysis, revealing three subpopulations represented by two Korean potato groups and one separate foreign potato group within 110 lines. When analyzing 393 global potato clones, country/region-specific genetic patterns were revealed. The Korean potato clones exhibited higher heterozygosity than those from Japan, the United States, and other potato landraces. We also employed integrated extended haplotype homozygosity (iHS) and cross-population extended haplotype homozygosity (XP-EHH) to identify selection signatures spanning candidate genes associated with biotic and abiotic stress tolerance. Based on the informativeness of SNPs for dosage genotyping calls, 10 highly informative SNPs discriminating all 393 potatoes were identified. Our results could help understanding a potato breeding history that reflects regional adaptations and distinct market demands.

## Introduction

As the world’s third most important food crop in terms of human consumption after wheat and rice^1^, the potato (*Solanum tuberosum* L.) is cultivated worldwide as a main crop, double crop, or intercrop. It is consumed fresh or in various processed forms, as well as having some industrial applications. In Korea, potatoes are grown commercially all over the country. According to data collected by the Rural Development Administration (RDA) in 2021, Gangwon province, a cool and mountainous region, grows more potatoes than any other province, followed by Gyeongbuk, Chungnam, and Jeju Island. The potato breeding programs at the Highland Agriculture Research Institute (HARI), located at an elevation of 800 m above sea level in Gangwon province, were established in 1961, aiming to provide improved local potato cultivars with high yield and quality. In recent years, emphasis has been placed on developing early maturing varieties with a short dormancy period for double cropping and chip processing potatoes, which would benefit farmers and expand their market share. After the large-scale cultivation of Namjak (a.k.a. Irish Cobbler) potatoes from the 1960s to early 1980s, introductions like Sumi (Superior) (released in 1978) and Daeji (Dejima) (released in 1978) as fresh table potatoes, and Deaseo (Atlantic) (released in 1995) as a chip processing potato, have been widely cultivated in Korea^2^. The local potato varieties released over the past 20 years, such as Chubaek, Golden Ball, and Mangang, have been good alternatives to the previously cultivated foreign potatoes in terms of their early marketability and outstanding processing properties. Although the HARI Potato Breeding program has developed and released 28 cultivars, including 26 clonal selections, there is a need to develop new varieties which meet demands for high yields under low inputs,resistance against diseases and pests such as potato virus Y, late blight (*Phytophthora infestans*), and common scab (*Streptomyces scabies*),tolerance to abiotic stresses like high temperatures; favorable processing properties such as cold sweetening tolerance and long dormancy in storage; and improved health and nutritional properties.

Breeding potatoes remains challenging because the complexity of their auto-tetraploidy and highly heterozygous genome, the complexity of their plant and crop physiology, the duration of their growth cycle, their low multiplication factor and the difficulties with the evaluation of their phenotype, have all resulted in slow progress, compared with diploid plants such as Arabidopsis, rice and tomato^3^.

Revealing potato genetic diversity and population structure is an essential step for breeding efforts, which involves identifying promising parental combinations from the germplasm collections, crossing the parents to generate genetic variation, and selecting clones with target traits. Although, in the genomics era, molecular breeding of polyploidy crops such as potato has lagged behind many diploid crop species, genetic evaluation of potato clones has been conducted using various molecular markers available (reviewed by^4^, for example, random amplified polymorphic DNA (RAPD), amplified fragment length polymorphism (AFLP), inter simple sequence repeats (ISSR), inter-retrotransposon amplified polymorphism (IRAP) and simple sequence repeats (SSR). With the advantages of abundance, cost-efficiency, and high-throughput assays, single nucleotide polymorphism (SNP) markers have become increasingly important in crop genetic studies (reviewed by^5^. At present, the hybridization based SNP array and next generation sequencing (NGS) enabled genotyping such as genotyping by sequencing (GBS) are the most popular high throughput genotyping platforms.

Technically, two platforms, Illumina Infinium and Affymetrix Axiom, have been used for SNP array in polyploids. A major benefit of the SNP array for polyploids compared to GBS data is the ability to accurately determine allele dosage, but the cost of the array is determined by sales volume^6–8^.

In potato, high-throughput genotyping platforms^9–11^ that provide genome-wide representation of the single nucleotide polymorphisms (SNPs) present in the potato germplasm have been developed and applied to investigate genetic diversity and population structure. Four open access software tools have been developed and used for genotype calling in polyploidy crop species based on array data, including Illumina GenomeStudio (https://www.illumine.com/techniques/microarrays/), ClusterCall R package^7^, a web-based software SuperMASSA^12^, and fitTetra R package^6^. The genetic diversity of 250 diverse North American potatoes was characterized using an 8 K SNP array^10^. They performed STRUCTURE analysis using the diploid genotype calls to demonstrate clear differences between cultivated potato clones and related wild species, and a minimal substructure within the cultivated potatoes. Also, clear separation between potato market classes was observed with pairwise kinship estimates. Igarashi et al*.* genotyped 164 Japanese potatoes based on a diploid model using a 12 K SNP array and compared them with North American and European potatoes^13^.

Vos et al*.*^11^ designed a 20 K SolSTW array and analyzed it using fitTetra software to genotype a total of 569 potato clones and identified introgression segments, selection, and founder signatures. Pandey et al*.*^14^ investigated the genetic diversity and population structure of 214 potato advanced clones selected and maintained in vitro over a 40-year period by the Texas A&M University Potato Breeding Program with the Illumina Infinium 22 K V3 Potato Array. They performed STRUCTURE and discriminant analysis of principal components (DAPC) using diploid genotypic calls and hierarchical clustering (HC) using tetraploid genotypic calls to divide the clones into three clusters.

Plant domestication and artificial selection give rise to gradual changes in populations at the genomic level^15^. Subsequent footprints of selection, known as selection signatures or selective sweeps, can be traced in the genomes of many crop plants that have been subjected to breeding programs to improve traits of interest, such as yield, pest resistance, and flesh color. Pandey et al*.*^14^ carried out a selection signature analysis using the PCAdapt, iHS, and XP-EHH approaches to identify candidate genes controlling potato flesh and skin color, length of plant cycle and tuberization, and carbohydrate metabolism.

Characterizing germplasm identity and purity is an essential component of breeding and germplasm management^16^. Recently, SNP quality assurance and control genotyping methods based on low-density SNPs have been investigated in maize^16^ and sweetpotato^17^. In sweetpotato, a 30 SNP-set with uniform distribution across chromosomes was selected to identify relatively similar mislabeling error rates as a high density SNP-set of 10,159 markers, while a minimum of 80 selected SNP markers was employed to distinguish each of the CIMMYT maize inbred lines (CMLs) entries from one another.

The objective of this study was to characterize the potato varieties and advanced breeding clones bred by Korean potato breeding programs at a genome-wide level in the context of global potato breeding history. To achieve this, a 110-line diversity panel that included the available local varieties and advanced clones, as well as foreign potatoes, was genotyped using 8 K SNP markers and dosage genotype calling methods to assess genetic diversity and population structure. Furthermore, we constructed a merged dataset using ClusterCall software to compare Korean potatoes with those bred by national potato breeding programs in Japan, the United States, Europe, and other countries, to illustrate how local/regional potatoes have evolved in relation to landrace potatoes (pre-1930). Based on the separation of the potatoes by country (Korea, Japan, and the United States), we employed selection signature analysis to identify candidate loci that could be associated with local adaptations. The percent heterozygosity of the clones was calculated to reveal higher heterozygosity for Korean potatoes. Based on calculations of the informativeness for dosage genotype calls, 10 SNP markers were selected that could identify all 393 clones used in this study.

## Results

### Population structure analysis of the 110-line Korean potato germplasm panel using STRUCTURE, DAPC, and HC

STRUCTURE analysis provided an estimation of the number of populations in the Korean potato germplasm panel. The estimation of the delta K value, using Evanno's method, showed the highest peak at K = 3 (Fig. S3), indicating that the 110 clones in the panel could be grouped into three clusters based on differences in their genetic makeup. For the DAPC analysis, the lowest Bayesian information criterion (BIC) value of K = 3 was obtained using the *find.clusters* function, confirming structured population, except no admixture clones (Table S9, Fig. S4).

**Figure 1:**
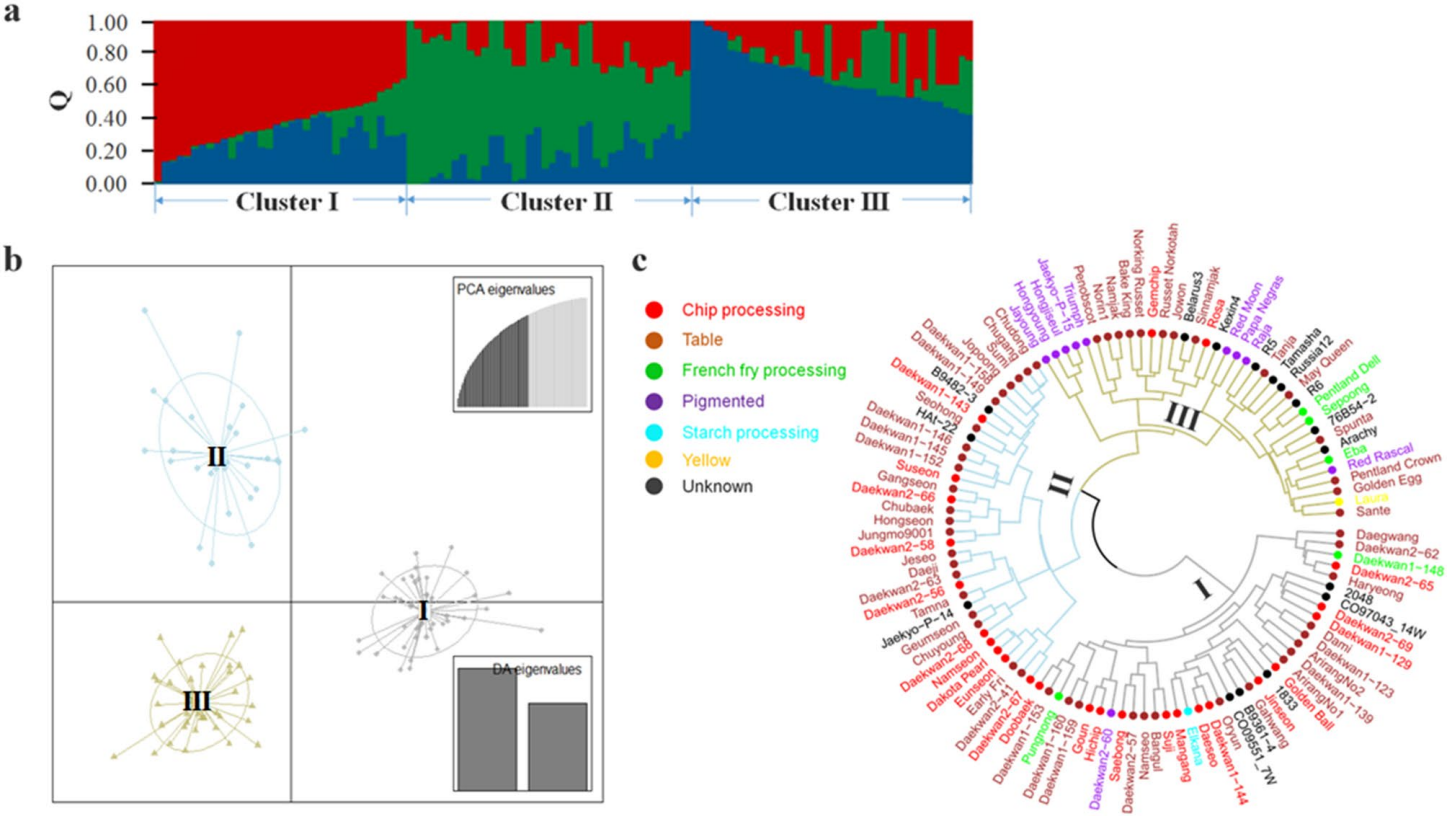
The 110-line Korean potato germplasm consists of three subgroups which were inferred using three different approaches, STRUCTURE, discriminant analysis of principal components (DAPC), and hierarchical clustering (HC). Most of the Korean potatoes grouped together into two clusters, whereas the foreign potatoes were placed into the third cluster. (**a**) Proportional membership (Q) of each clone showing three distinct clusters using 6575 SNP markers. (**b**) DAPC using the adgenet R package confirmed the structured population. The axes represent the first two linear discriminants and the small solid dots and ellipses represent each clone. The numbers in the circles indicate the different subpopulations identified by DAPC analysis. (**c**) A dendrogram of the 110 clones using HC (method = “ward.D2”). Two major clusters are observable, in which one cluster indicates I and another one consists of two subgroups (II and III). Note that Cluster III is presented in dark khaki, Cluster II in light blue, and Cluster I in dark gray, corresponding to the colors of the subgroups inferred by DAPC. The leaf colors indicate the respective market class of the individual clones.

The Ward dendrogram generated using Nei’s genetic distance and hierarchical clustering also revealed the presence of three clusters in the population represented by the 110 potato clones (Fig. 1, Table S9).

The individual clusters for STRUCTURE, DAPC, and HC constituted similar sets of clones. For example, the clusters that included cv. Namjak also included the majority of the foreign potato clones, 26 (70.3%), 26 (70.3%), and 28 (75.7%) of the 37 foreign clones, respectively. In the Cluster I (Namjak group), the average percentage of foreign clones that were common across all three population structure analyses was 87.9%. Six Korean potato varieties (Namjak, Sinnamjak, Golden Egg, Sepoong, Jayoung, and Hongyoung) were common in all three methods, and two more Korean potato clones (Hongjiseul and Jaekyo-P-15) were commonly present in the DAPC and HC results. Thus, the four colored potatoes (Jayoung, Hongyoung, Hongjiseul, and Jaekyo-P-15), which are pigmented in both their skin and flesh, were grouped together in the Cluster I by DAPC and HC (Table S9). The remaining Korean potatoes (over 86.5%) were divided into two clusters, in which either cv. Daeseo or cv. Sumi were present. In the Cluster II (Daeseo group), the average percentage of Korean potato clones that were common across all three population structure analyses was 75.3%, while that of the Cluster III (Sumi group) was 73.3%. DAPC showed the highest average percentage of varieties common to more than two methods across all three clusters, suggesting that the DAPC results could be more reliable than the STRUCTURE or HC results as described in^18,19^.

We also calculated population genetics parameters, some of which came from diploid genotype calls, dosage calls, or both (Table 1). The minor allele frequency (MAF) ranged from 0.05 to 0.50, with a mean of 0.28. It was calculated by snpReady in R using diploid genotype calls. This value was similar to that calculated using the function *minorAllele* in the adegent R package following the tetraploid model. We can easily calculate the number of transitions across the samples for the genotype calls of specific markers, so that the value indicates the informativeness of the SNP markers used in this study. Unlike the polymorphic information content (PIC), informativeness is calculated using dosage genotype calls. The PIC ranged from 0.08 to 0.38 with a mean of 0.30, whereas the informativeness ranged from 0.25 to 0.79 with a mean of 0.64. The average observed heterozygosity (0.51) calculated using diploid genotype calls was smaller than the average percent heterozygosity (0.63) calculated using dosage genotype calls. The average distance among the clones in the same cluster ranged from 0.32 to 0.37. The Cluster II showed the highest heterozygosity among the clones, indicating that it was highly diverse, whereas the other two clusters showed lower heterozygosity.

**Table 1:** Summary of population genetics parameters in the Korean potato germplasm panel with different single nucleotide polymorphism calling methods.

Parameter	Range	Value	Type of genotype call software used
Minor allele frequency (MAF)	Mean	0.28	diploid genotype calls
	Lower	0.05	snpReady R package
	Upper	0.50	
	Mean	0.27	dosage genotype calls
	Lower	0.05	adegenet R package
	Upper	0.50	
Polymorphic information content (PIC)	Mean	0.30	diploid genotype calls
	Lower	0.08	snpReady R package
	Upper	0.38	
Informativeness	Mean	0.64	dosage genotype calls
	Lower	0.01	this study
	Upper	0.87	
Ho (observed heterozygosity)	Mean	0.51	diploid genotype calls
	Lower	0.31	snpReady R package
	Upper	0.74	
	Mean	0.63	dosage genotype calls
	Lower	0.42	percent heterozygosity
	Upper	0.79	
Average distances between clones in the same cluster	Namjak group	0.32	dosage genotype calls
	Daeseo group	0.37	STRUCTURE2.3.4
	Sumi group	0.32	
Mean fixation index (F_ST_)	Namjak group	0.13	dosage genotype calls
	Daeseo group	0.07	STRUCTURE2.3.4
	Sumi group	0.18	
TajimaD		3.37	diploid genotype calls
			TASSEL
		4.04	dosage calls converted to diploid calls
			TASSEL

The fixation index (F_st_) measures the genetic distance between populations. The Cluster III had the highest F_st_ value (0.18), while the Cluster II had the lowest (0.07), indicating that the clones in the former group are not currently breeding with one another, whereas those in the latter group share their genetic material through high levels of breeding. Tajima’s D statistic was used to compare the observed nucleotide diversity against the expected diversity under the assumptions of selectively neutral polymorphisms and a constant population size^20^. The value (3.37) of Tajima’s D using diploid genotype calls was smaller than that (4.04) obtained after converting the dosage forms (AAAA, AAAB, AABB, ABBB, and BBBB) into diploid forms (AAAA = AA, BBBB = BB, and AAAB, AABB, ABBB = AB) for use in analysis packages that do not support polyploid data. The value of 4.04 is close to that (4.29) obtained in a diploidized version described by Pandey et al.^14^.

### DAPC, HC, and KLFDAPC analyses for an extended genetic diversity panel

We further investigated the Korean potato clones in the Korean potato germplasm panel using an extended genetic diversity panel that included 94 Japanese potatoes^13^, 164 American potatoes, 15 Canadian potatoes, two German potatoes, one Chilian potato, and three potatoes of unknown origin^10^. The ClusterCall R package was used to obtain dosage genotype calls from the XY raw data of the Japanese potatoes, the publicly available theta data of the potato clones from North America and other countries^7^, and the .idat data from the Korean potato germplasm panel. Subsequently, the three dosage genotype calls were merged into a single dataset, hereafter referred to as the extended genetic diversity panel, based on common SNP markers. After filtering with the criteria MAF = 0.05 and call rate = 0.90, 3977 SNP markers remained (Table S3). DAPC was performed and the lowest Bayesian information criterion value was found to be 6 (Fig. S5). Similar to the DAPC analysis using the Korean potato germplasm panel, the DAPC analysis divided the Korean potatoes into two well-defined clusters according to their genetic structure, one group containing cv. Daeseo and the other cv. Sumi. Interestingly, when these clusters were compared with the 110-line panel DAPC clusters, almost all the admixed clones (18 of 20), determined by the 110-line panel STRUCTURE analysis, moved from the Cluster II (where they appeared in the 110-line panel analysis) to the Cluster III (in the 393-line extended panel analysis, Table S10). The four flesh-colored potatoes, Jayoung, Hongyoung, Hongjiseul, and Jaekyo-P-15, moved from the Cluster I to the colored group (Table S10).

**Figure 2:**
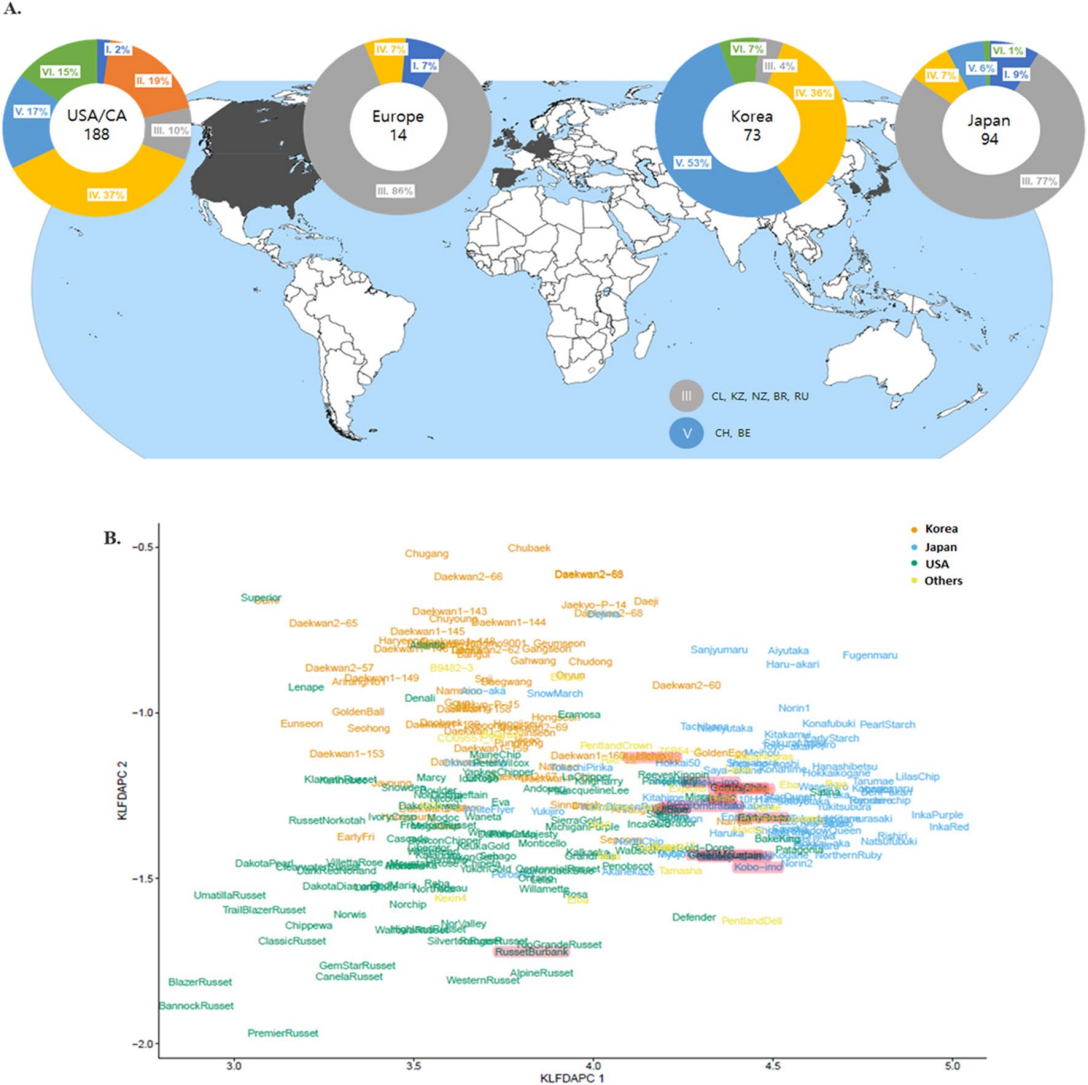
A potato genetic landscape revealed by DAPC and KLFDAPC. (**A**) The ringplot shows the percentages of clones belonging to each of the six inferred clusters based on DAPC for an extended 393-line diversity panel. . The world map was downloaded from the Wikipedia (https://en.wikipedia.org/wiki/File:World_Map_Blank_-_with_blue_sea.svg). The percentages were calculated for 188, 14, 73, and 94 potato clones from North America, Europe, Korea, and Japan, respectively. Note that clones with unknown origins were not included in the ringplots. Below the world map are the clusters originating from the seven countries from which only one potato clone was analyzed in this study. The roman numbers represent individual clusters identified by DAPC. CL; Chile, KZ; Kazakhstan, NZ; New Zealand, BR: Brazil, RU; Russia, BE; Belarus, CH; China. (**B**) Population genetic structure projected by the first two reduced features in KLFDAPC with σ = 2 for the Korean potato clones and potato varieties released from Japan, the United States, and other countries (Table S2). These results confirm that clustering depends on the geographical location (Korea, Japan, and the USA) where the original crossing was carried out. Potato clones from Europe and other countries are placed into the Japanese cluster. The landrace potatoes are highlighted.

The DAPC analysis of the extended potato diversity panel using 3977 SNP markers showed that differences in the percentages of the potato clones in specific clusters clearly reflect their country/regional origins. Figure 2A shows a ring plot representing the percentage of clones assigned to the six inferred clusters based on DAPC. For the 73 Korean potatoes, 36% were grouped into Cluster IV and 53% were assigned to Cluster V; altogether, 89% of the Korean potatoes were grouped into these two clusters. The 54% North American potatoes were placed into Clusters IV and V. The potatoes in Clusters II and VI were the Russet (19%) and pigmented (15%) potatoes, respectively. The Russet class was unique across all countries. Moreover, 77% of the 94 Japanese potatoes and 86% of the 14 European potatoes were grouped into Cluster III. Although only one potato clone each was analyzed from Chile, Kazakhstan, New Zealand, Brazil, and Russia, they also grouped together into Cluster III (Fig. 2A). Two clones, originating from China and Russia, were assigned to Cluster V. We also performed kernel local fisher DAPC (KLFDAPC), a nonlinear version of DAPC, which could rectify the limitations of linear approaches by preserving nonlinear information and the multimodal space of the samples^21^. The population genetic structure was projected by the first two reduced features of the KLFDAPC with σ = 2, for the Korean potato clones and the potato varieties released from Japan, the United States, and other countries (Fig. 2B). This confirmed that clustering depended on the geographical location (Korea, Japan, and the USA) where the original crossing was carried out. Potato clones from Europe and other countries were placed in the Japanese clusters. Interestingly, the potato landraces highlighted in Fig. 2B overlapped three different groups from Korea, Japan, and the United States. It is likely that the clear distinction between the American potatoes and Korean/Japanese clones was caused by the Russet varieties.

The HC for the extended panel using 3977 SNP markers showed clustering profiles similar to those of the DAPC. The HC dendrogram (Fig. S6) led to an easily recognizable visualization of the duplicates among the 393 clones, whose pairwise genetic distances were zero or almost zero. The identified duplicates were Namjak vs. Irish Cobbler, Sumi vs. Superior, Daeseo vs. Atlantic, Daeji vs. Dejima, CO97043_14 W vs. MSQ070-1, Rosa_hari vs. Rosa, Russet Norkotah_hari vs. Russet Norkotah-S8 vs. Russet Norkotah-S3, Norin1_hari vs. Norin1, and InkaRouge_2x vs. Inka-no-mezame_2x. The former potatoes were from the 110-line panel and the latter from the 393-line extended panel.In fact, the Korean varieties Namjak, Daeseo, Sumi, and Daeji, in the 110-line panel are introduced and renamed from abroad as the cultivars, Irish Cobbler (Unknown), Atlantic (USA), Superior (USA) and Dejima (Japan), respectively. In addition, the foreign potatoes (Rosa_hari, Russet Norkotah_hari, and Norin1_hari) in the 110-line panel, were placed beside the original varieties from the extended panel with genetic distance = 0, indicating that the potato clones maintained in Korea have the same genetic identity as the original ones.

InkaRouge_2x and Inka-no-mezame_2x were duplicated, as described by Igarashi et al*.*^13^. The HC dendrogram showed the chip processing market potatoes grouped together, as were the pigmented potatoes and Russet varieties.

### Heterozygosity and informativeness for a 393-line extended genetic diversity panel

**Figure 3:**
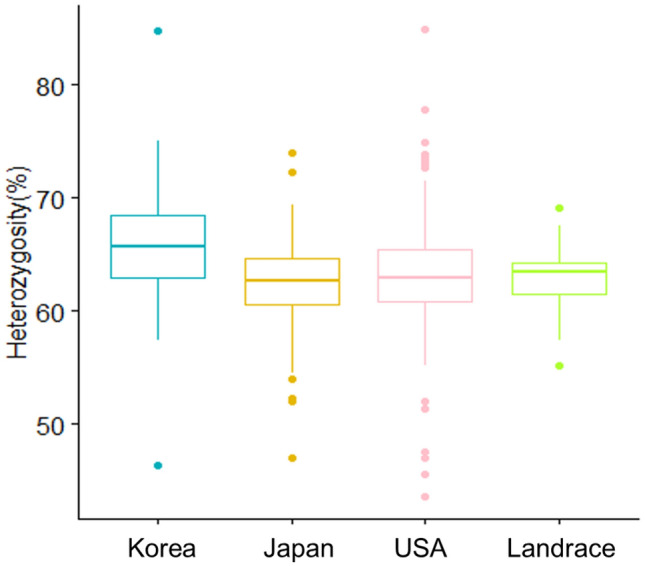
A boxplot showing the genome-wide percent heterozygosity for four populations (Korea, Japan, the USA, and landraces). Note: 2 × varieties were excluded in the Japanese population.

The percentage of heterozygous SNP loci (percent heterozygosity) for the 393 lines is shown in Fig. 3. The percent heterozygosity for 68 (93.2%) of the 73 Korean potato clones was > 60.0% (Table S11). The highest percent heterozygosity was observed in cv. Daeseo (a.k.a., Atlantic), as described by Igarashi et al*.*^13^.

The Korean potato clones exhibited a higher average percent heterozygosity (65.6%) than the clones from Japan, the United States, and other landraces potatoes (62.4%, 63.2%, and 62.9%, respectively) according to a non-parametric Wilcoxon test (*P* < 0.001).

The informativeness of the 3977 SNP markers for the 393 potato clones from Korea, Japan, the United States, and other countries was calculated based on the transitions of genotype calls across samples, ranging from 25.4 to 79.4% (Table S12). The use of ten highly informative SNP markers could identify all 393 clones used in this study, including the duplicate clones (Fig. S7), being a power of discrimination equal to a high density SNP-set of 3977 markers. The MAF values for the selected 10 SNP-set were ≥ 0.40 except two markers (Table 2).

**Table 2:** The selected 10 SNP-set discriminating 393 potato clones used in this study.

No	Marker	The percent informativeness (%)	Chromosome	Position	SNP	MAF
1	solcap_snp_c2_40883	79.4	9	51,989,783	[T/C]	0.42
2	solcap_snp_c1_4078	79.1	9	46,078,957	[A/G]	0.45
3	solcap_snp_c2_36385	78.9	6	1,461,571	[A/G]	0.40
4	solcap_snp_c2_25179	78.1	2	40,294,533	[A/G]	0.50
5	solcap_snp_c2_22095	78.1	9	53,969,586	[A/C]	0.43
6	solcap_snp_c2_35705	77.6	2	47,327,646	[T/C]	0.37
7	solcap_snp_c2_19081	77.6	8	52,490,785	[A/G]	0.48
8	solcap_snp_c1_9027	77.6	10	56,116,729	[A/G]	0.49
9	solcap_snp_c1_513	77.4	7	43,427,122	[A/G]	0.20
10	solcap_snp_c1_2786	77.4	7	25,775,384	[A/C]	0.47

### Detection of SNP loci under selection

**Figure 4:**
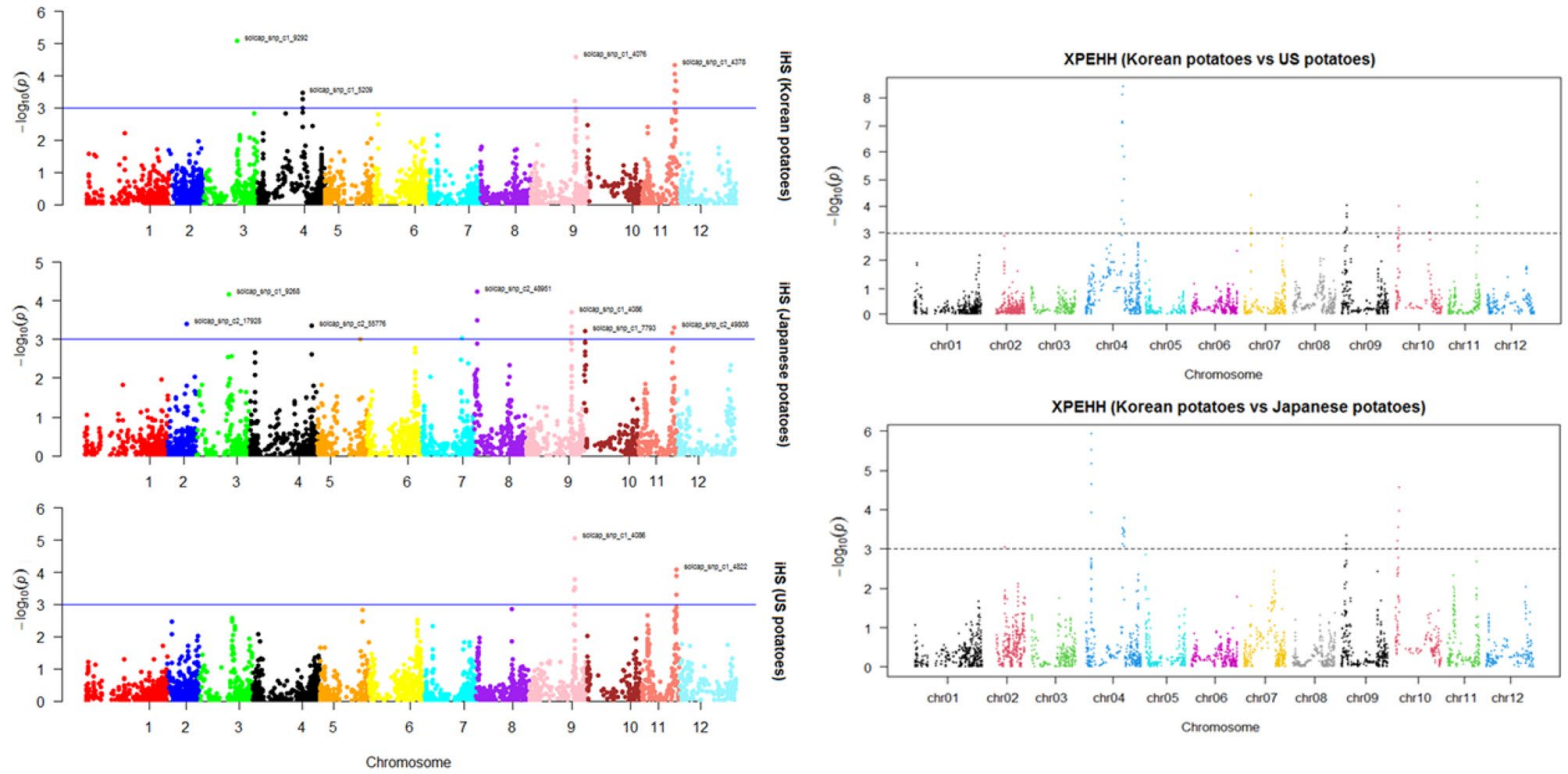
Manhattan plot of the genomic regions detected by integrated extended haplotype homozygosity (left) and cross-population extended haplotype homozygosity (XP-EHH, right) as being under putative selection. The solid/dashed lines represent the significant threshold level for − log_10_ (p-value).

A total of 70 SNP loci under selection were identified using iHS and XP-EHH (Fig. 4, Table S13), among which the 13 top significant SNPs detected by both approaches are shown in Table 3, along with the putative functions of the candidate genes containing these significant SNPs. Candidate genes spanning ~ 100 kb upstream and downstream of top significant SNPs were retrieved (Table 4), revealing that the Korean potatoes have footprints associated with several genes essential for biotic and abiotic stress tolerance.

**Table 3:** Candidate genes containing top significant single nucleotide polymorphisms detected using integrated extended haplotype homozygosity and cross-population extended haplotype homozygosity analyses.

Top significant SNP	chr	Candidate gene	Putative function	Detection analysis	Max statistic	Log p-value
solcap_snp_c1_5209	4	*Soltu.DM.04G019230*	D-aminoacid aminotransferase-like PLP-dependent enzymes superfamily protein	iHS_sk	3.6	3.5
				XPEHH_sk_us	4.0	4.2
solcap_snp_c2_16722	4	*Soltu.DM.04G020490*	Protein Ycf2	XPEHH_sk_us	5.4	7.1
				XPEHH_sk_jp	3.4	3.1
solcap_snp_c2_16712	4	*Soltu.DM.04G020520*	Conserved hypothetical protein	iHS_sk,	3.3	3.0
				XPEHH_sk_us	5.4	7.1
				XPEHH_sk_jp	3.5	3.4
solcap_snp_c2_16718	4	*Soltu.DM.04G020610*	Cytochrome P450, family 71, subfamily A, polypeptide	iHS_sk	3.5	3.3
				XPEHH_sk_us	5.8	8.1
				XPEHH_sk_jp	3.6	3.5
solcap_snp_c2_16744	4	*Soltu.DM.04G020750*	Cystathionine beta-synthase (CBS) family protein	XPEHH_sk_us	3.6	3.5
				XPEHH_sk_jp	5.9	8.4
solcap_snp_c1_14442	4	*Soltu.DM.04G021160*	Phosphotyrosine protein phosphatases superfamily protein	XPEHH_sk_us	4.8	5.8
				XPEHH_sk_jp	3.8	3.8
solcap_snp_c1_16534	4	*Soltu.DM.04G021190*	C2H2 and C2HC zinc fingers superfamily protein	XPEHH_sk_us,	4.4	5.0
				XPEHH_sk_jp	3.6	3.6
solcap_snp_c2_39856	4	*Soltu.DM.04G021220*	Ubiquitin-specific protease	XPEHH_sk_us	3.5	3.3
				XPEHH_sk_jp	3.5	3.4
solcap_snp_c1_4271	9	*Soltu.DM.09G006270*	Phosphatidic acid phosphatase (PAP2) family protein	XPEHH_sk_us	3.7	3.7
				XPEHH_sk_jp	3.4	3.1
solcap_snp_c2_13242	9	*Soltu.DM.09G006310*	IQ-domain	XPEHH_sk_us	3.9	4.0
				XPEHH_sk_jp	3.5	3.3
solcap_snp_c1_4248, solcap_snp_c2_13194	9	*Soltu.DM.09G006540*	Leucine-rich repeat protein kinase family protein	XPEHH_sk_us	3.7	3.6
				XPEHH_sk_jp	3.3	3.0
solcap_snp_c1_4076	9	*Soltu.DM.09G018820*	GroES-like zinc-binding dehydrogenase family protein	iHS_sk	4.2	4.6
				XPEHH_sk_us	3.2	2.9

**Table 4:** The candidate selective sweep regions around the most significant single nucleotide polymorphisms, identified using integrated extended haplotype homozygosity and cross-population extended haplotype homozygosity analyses, which are associated with biotic or abiotic stress tolerances.

Top significant SNP	Selective sweep region	Candidate gene	Putative function
solcap_snp_c1_5209	chr04:43,730,218–43,937,038 (206.82 Kb)	*Soltu.DM.04G019240*	Nuclear factor Y, subunit B1
solcap_snp_c2_16722, solcap_snp_c2_16712	chr04:47,061,351–47,267,899 (206.55 Kb)	*Soltu.DM.04G020470*	GTP-binding family protein
solcap_snp_c2_16718	chr04:47,428,515–47,632,604 (204.09 Kb)	*Soltu.DM.04G020570*	Cytochrome C1 family
		*Soltu.DM.04G020580*	LRR and NB-ARC domains-containing disease resistance protein
		*Soltu.DM.04G020590*	DUF4228 domain-containing protein
		*Soltu.DM.04G020610*	Cytochrome P450, family 71, subfamily A, polypeptide
solcap_snp_c2_16744	chr04:47,735,961–47,943,799 (207.84 Kb)	*Soltu.DM.04G020730*	Non-intrinsic ABC protein
		*Soltu.DM.04G020740*	Leucine-rich repeat (LRR) family protein
		*Soltu.DM.04G020750*	Cystathionine-β-synthase (CBS) family protein
		*Soltu.DM.04G020760*	Auxin efflux carrier family protein
		*Soltu.DM.04G021140*	WRKY DNA-binding protein
		*Soltu.DM.04G021150*	Zinc finger (C2H2 type) family protein
		*Soltu.DM.04G021160*	Phosphotyrosine protein phosphatases superfamily protein
		*Soltu.DM.04G021160*	Tyrosine phosphatase family protein
solcap_snp_c1_16534	chr04:48,682,426–48,885,011 (202.59 Kb)	*Soltu.DM.04G021170*	AIG2-like (avirulence induced gene) family protein
		*Soltu.DM.04G021190*	C2H2 and C2HC zinc fingers superfamily protein
		*Soltu.DM.04G021200*	Myb domain protein
		*Soltu.DM.04G021220*	Ubiquitin-specific protease
		*Soltu.DM.04G021240*	Calmodulin-binding transcription activator protein with CG-1 and ankyrin domains
		*Soltu.DM.09G006200*	PLAC8 family protein
		*Soltu.DM.09G006320*	Chaperone DnaJ-domain superfamily protein
		*Soltu.DM.09G006330*	Alpha/beta-hydrolases superfamily protein
		*Soltu.DM.09G006410*	Ascorbic acid mannose pathway regulator
		*Soltu.DM.09G006420*	F-box family protein with a domain of unknown function (DUF295)
		*Soltu.DM.09G006440*	ROP interactive partner
		*Soltu.DM.09G006490*	Minichromosome maintenance
		*Soltu.DM.09G006530*	Nuclear factor Y subunit B10
		*Soltu.DM.09G006540*	Leucine-rich repeat protein kinase family protein
		*Soltu.DM.09G006560*	Ascorbate peroxidase
		*Soltu.DM.09G006620*	Zinc finger CCCH-type family protein, an effective role in stress tolerance
		*Soltu.DM.09G006630*	Tetratricopeptide repeat (TPR)-like superfamily protein, involved in plant hormone signaling
solcap_snp_c1_4076	chr09:52,445,372–52,651,048 (205.68 Kb)	*Soltu.DM.09G018750*	pfkB-like carbohydrate kinase family protein, involved in the pathway starch biosynthesis
		*Soltu.DM.09G018760*	Protein kinase superfamily protein
		*Soltu.DM.09G018770*	Transducin family protein/WD-40 repeat family protein
		*Soltu.DM.09G018820*	GroES-like zinc-binding dehydrogenase family protein
		*Soltu.DM.09G018830*	Tetratricopeptide repeat (TPR)-like superfamily protein
		*Soltu.DM.09G018840*	RPM1 interacting protein, essential regulator of plant defense

For example, candidate genes encoding the RPM1 interacting protein (*Soltu.DM.09G018840*), an essential regulator of plant defense, and leucine-rich repeat (LRR) family proteins (*Soltu.DM.04G020580*, *Soltu.DM.04G020740*, *Soltu.DM.09G006540*) were identified, whereas candidate genes such as nuclear factor Y (*Soltu.DM.04G019240*), the cystathionine beta-synthase family protein (*Soltu.DM.04G020750*), the zinc finger CCCH-type family protein (*Soltu.DM.09G006620*), and ascorbate peroxidase (*Soltu.DM.09G006560*) were identified for abiotic stress tolerance (Table S13).

## Discussion

In this study, we used genome-wide SNP markers to evaluate a diversity panel composed of 393 potato varieties and advanced breeding lines that have been bred by different breeding programs worldwide, particularly from Japan, the United States, and Europe, focusing on their comparisons with Korean potatoes. The 110-line diversity panel, which included 45 commercial cultivars and 28 advanced breeding clones bred by Korean potato breeding programs, as well as 37 foreign potatoes, was investigated using three different complementary approaches: STRUCTURE, DAPC, and HC. The Korean potatoes were divided into two groups, represented by cvs. Sumi and Daeseo, in agreement with the wide use of one of parents for cross breeding (Table S1). In the past, many foreign varieties were introduced and tested in local Korean environments. However, only a few varieties have been cultivated. For example, the potato varieties Atlantic, Superior, Irish Cobbler, and Dejima have been introduced and released under the registered names Daeseo, Sumi, Namjak, and Daeji, respectively^2^. When analyzing the Korean potatoes in the 393-line diversity panel, it was clear that they grouped together according to their market class (Fig. S6). For example, Korean potatoes suitable for chip processing were grouped together with foreign chip processing potatoes and the colored potatoes, cvs. Hongyoung, Jayoung, Hongjiseul, Jaekyo-P-15, and Daekwan2-60, were placed in the pigmented group that included Red Maria, Chieftain, All Blue, Shadow Queen, Purple Majesty, Winema, Aino-aka, Dragon Red, etc. We also looked at the groupings of landrace potatoes (pre-1930) (Triumph, Garnet Chile, Purple Peruvian, Nemuromurasaki, Kintoki-imo, Green Mountain, Russet Burbank, May Queen, Early Rose, Benimaru, and Kobo-imo) in the HC analysis (Fig. S6) and the KLFDAPC. In the KLFDAPC, they were placed centrally, overlapping the more recently bred potatoes from Korea, Japan, and the United States in different directions, visually supporting the history of potato breeding and how potato varieties have diversified according to various breeding strategies (Fig. 2B).

Among the various potato types grown in the United States, the Russet potato is the most popular market class^22^. Russet potatoes are unique to the United States, and are not selected by breeding programs in either Korea or Japan, taking consumers’ preferences into account. Approximately 35% of the potatoes in Japan are used for starch production^13^ and many modern Japanese varieties have T-type chloroplast DNA^13,23^, supporting the result that Japanese potatoes were not differentiated from European ones in our study. Unfortunately, most European varieties do not perform well in Korean environments. Korean potato programs have been pursuing the development of diverse market class potatoes, such as potatoes suitable for chip processing, French fries, and double cropping (spring/summer season products are used as seeds for winter season production in the south) under low input conditions. Accordingly, several promising varieties have been developed and released for agricultural deployment as alternatives to foreign varieties such as Daeseo (Atlantic) or Sumi (Superior). In terms of the high heterozygosity of Korean potatoes (Fig. 3 and Table S11), it might be wise to direct breeding efforts to improve Atlantic potatoes to adapt well to local environmental conditions, as they showed the highest genome-wide percent heterozygosity of the studied varieties and are the most popular variety grown worldwide^13^.

Regarding the approaches employed in this study to reveal the genetic diversity and population structure of cultivated potatoes, dosage genotype calls could lead to more reasonable and accurate results than diploid genotype calls (Table 1). If no packages that support polyploid data are available, biallelic markers could be called in a diploidized version which means that the three heterozygous classes expected in potato were converted into one heterozygous class^17,24^. The use of appropriate methods for integrating different sources of SNP data could result in biologically meaningful outcomes, because previously, we recognized “strange” outcomes when we simply merged the publicly available genotype datasets (data not shown).

We identified several candidate genes, with 3977 SNP markers, related to biotic and abiotic stress tolerance that may be involved in adaptation to local environmental conditions. Candidate genes with putative functions, such as the RPM1 interacting protein (*Soltu.DM.09G018840*), LRR/NB-ARC domain-containing disease resistance proteins (*Soltu.DM.04G020580*, *Soltu.DM.04G020740*, *Soltu.DM.09G006540*), nuclear factor Y (*Soltu.DM.04G019240*), the zinc finger CCCH-type family protein (*Soltu.DM.09G006620*), and ascorbate peroxidase (*Soltu.DM.09G006560*), were identified. RPM1-interacting protein 4 (RIN4) is a conserved plant immunity regulator that has been extensively studied and can be modified by pathogenic effector proteins^25^. RIN4 plays an important role in both pattern triggered immunity and effector-triggered immunity. Most disease resistance genes in plants encode nucleotide-binding site LRR proteins^26^. The nuclear factor Y complex plays multiple essential roles in plant growth, development, and stress responses^27^. CCCH genes are involved in plant developmental processes and biotic and abiotic stress responses^28,29^. The less-common CCCH type of zinc finger superfamily proteins are important in plant development and tolerance to abiotic stresses such as salt, drought, flooding, cold temperatures, and oxidative stress^29^. Ascorbate peroxidase catalyzes the conversion of H_2_O_2_ generated under environmental stress into H_2_O,therefore, it is of great importance as a key antioxidant enzyme in maintaining cellular homeostasis^30^. Although some important candidate genes were detected under selection, it is worth mentioning that the genome coverage of the current 8 K SNP array may be low, resulting in a lack of information on some important genomic regions harboring selection signatures. This issue may be addressed by using a greater density of SNPs.

In terms of methods to enable selection of a small number of SNP markers for the evaluation of germplasm identity and purity, we invented the number of transitions across the samples for the genotype calls of specific markers, rather than the use of the previously described selection criteria such as high minor allele frequency, sampling of clustered SNP in proportion to marker cluster distance and a uniform genomic distribution^16^. Our method enabled direct selection of the most informative SNPs with high minor allele frequency from the filtered high quality SNPs of 3977 without any considerations. The selected 10 SNP-set can be used to evaluate genetic identity, genetic purity, parent–offspring identity, and the validation of crosses in nurseries^16,17^. The identified SNP markers will be converted into a competitive allele-specific PCR (KASP) system and validated for routine use in breeding programs as well as germplasm conservation.

Overall, these results on the molecular characterization of cultivated potato clones could help understand how potato cultivars diversify for distinct market classes depending on each countries’ breeding strategies and could assist in genomics-facilitated breeding efforts to create new varieties that are better adapted to climate change and meet market demands.

## Materials and methods

### Plant materials

The germplasm used in this study comprised 110 diverse potato clones, including 73 Korean potato clones (45 commercial varieties and 28 advanced breeding lines) selected over 40 years by a potato breeding program in Korea, and 37 potato collections from various countries (Japan, the United States, the Netherlands, Germany, Spain, the UK, Russia, Belarus, Kazakhstan, Brazil, New Zealand, and China) (Table S1). Although nine of the foreign clones had an unknown origin, they were selected for this study according to their agronomic performance. All potato clones are available as tissue culture plants or tubers for field evaluations at Highland Agriculture Research Institute, National Agrobiodiversity Center, Rural Development Administration in Korea. Plant materials has been obtained and all experimental protocols in the present study complies with international, national, and institutional guidelines.

### SNP genotyping

The 110 lines in the Korean potato germplasm panel were genotyped using the Infinium 8303 Potato Array^9^, according to the manufacturer’s protocol (Insilicogen Inc., Gyeonggi-do 16,954, Korea). DNA was extracted from young leaf tissue from individual tissue culture plants, greenhouse-grown plants, or field plants using the QIAGEN DNeasy Plant Mini Kit (QIAGEN, USA), quantified using the DeNovix® DS-11 + Spectrophotometer (DeNovix Inc.), and adjusted to a concentration of 50 ng‧μL^−1^.

The data were analyzed using Illumina GenomeStudio software according to the GenomeStudio® Polyploid Genotyping Module v2.0 Software Guide (Illumina, San Diego, CA). The SNP genotype data were filtered to exclude SNPs that were monomorphic, had > 10% missing data, or mapped to duplicate places in the genome. In addition, the genotype data were filtered using < 0.05 MAF, calculated by the function *minorAllele* in the R package adegenet^31^. After filtering, 6575 SNPs remained (Table S3) and were distributed across the 12 chromosomes (Fig. S2). In addition, genotypes in nucleotide format were obtained in GenomeStudio, and a tetraploid format STRUCTURE input file (Table S4) was produced using a custom Python script. To determine the market class, phenotypic evaluations including tuber shape, tuber sucrose/glucose concentration, and chip color were carried out as described by Hirsch et al.^10^.

### Comparisons of reproducibility of dosage genotype calling methods

The three software packages, GenomeStudio (Illumina software), ClusterCall (R package)^7^, and polyBreedR (the function *geno_call*, R package) (https://polyploids.r-universe.dev/articles/polyBreedR/Vignette1.html), which have been developed to generate dosage genotype calls based on different models, were compared in terms of reproducibility for three independent replicates of the 16 Korean varieties (Table S5).

The average number of loci with contradicting calls within these replicates after filtering (call rate 0.90, MAF 0.05) was only 0.2%, with a maximum of 0.3%, in GenomeStudio, whereas in ClusterCall, the number of markers with discordant calls between replicates was only 0.4%, with a maximum of 0.8%. There were no significant differences between the two software programs.

In contrast, for the function *geno_call* of polyBreedR, which employed the normal mixture model implemented in the R package fitPoly, the average difference was 3.8%, with a maximum of 6.3%. Thus, ClusterCall was used to generate dosage genotype calls for the merged dataset from different sources of raw data, as described below (Table S5, Fig. S1).

### Merging datasets from Korea, Japan, the United States, and other countries

For the Japanese dataset, the XY data of 94 potatoes, including 88 Japanese varieties, four Japanese landraces, and two Japanese advanced breeding lines^13^ were kindly provided by Prof. Kazuyoshi Hosaka of the Potato Germplasm Enhancement Laboratory, Obihiro University of Agriculture and Veterinary Medicine, Obihiro, Hokkaido. The theta data for the United States and other countries (Canada, Germany, the Netherlands, the UK, and Chile) were from publicly available SNP data produced by Schmitz Carley et al.^7^. The XY data were converted to theta data, and ClusterCall software was used to obtain the dosage genotype calls. For the Korean dataset, in.idat format, dosage calls were also obtained using ClusterCall. The three dosage genotypes were merged into a single dataset based on the common markers across each dataset and were used for downstream analyses. The market class designations in the merged dataset were as described by Hirsch et al.^10^ and Igarashi et al.^13^.

### Genotype data analysis

#### Genetic diversity

The dosage genotype call data were used to study the genetic diversity. The MAF was calculated in the adegenet package^31^ in R^32^ and the allele frequency divergence among the clusters, fixation index, and average distance among individuals in the same cluster were calculated using STRUCTURE v2.3.4^33,34^. Tajima’s D was computed in TASSEL v5.0^35^ using modified genotype calls, which were obtained by converting the dosage forms (AAAA, AAAB, AABB, ABBB, and BBBB) into diploid forms (AAAA = AA, BBBB = BB, and AAAB, AABB, ABBB = AB). To compare the genetic diversity parameters of the dosage calls with those of the diploid calls, the allele frequency, PIC, heterozygosity, and inbreeding coefficient were calculated in the snpReady^36^ package in R, using the diploid genotype calls (Table S6).

#### STRUCTURE analysis

Population structure was determined using STRUCTURE software^33,34^ with an admixture model and five cluster-dosage genotype calls. Three replicates were performed for each value of K (number of populations) from 1–10 with a burn-in time and the number of Markov Chain Monte Carlo replicates after burn-in set to 10,000. The optimal number of subpopulations was obtained based on the delta K value calculated by the Evanno method using STRUCTURE HARVESTER^37,38^.

#### Discriminant analysis of principal components

The adegenet package^31^ in R was used to identify and describe clusters based on genetic relationships from tetraploid genotyping data. The *find.clusters* function was used to identify the number of clusters within the population.

#### Hierarchical clustering

Pairwise Nei’s genetic distance^39^ between clones was calculated with the StAMPP package^40^, using the dosage SNP genotype calls (Table S8). The resulting matrix was used to build a dendrogram using HC (method = “ward D2”) implemented in the dendextend^41^ and circlize packages^42^ in R.

### Kernel local fisher discriminant analysis of principal components analysis (KLFDAPC)

The dataset used for KLFDAPC analysis can be found in Table S7. The KLFDAPC package^21^ in R was used to obtain the pre-processed data containing the computed 10 principal components, followed by computing the kernels of local genetic features. The σ values of the KLFDAPC were adjusted to change the shape of the first two reduced features.

### Identification of selection signatures

Selection signature analyses were carried out using the filtered high quality 3977 SNPs for a total of 263 potato clones from Korea, Japan and the United States in the 393-line extended genetic diversity panel (Table S13) by applying two complementary statistical methods, the integrated haplotype homozygosity score (iHS)^43^ and the cross-population extended haplotype homozygosity (XP-EHH)^44^. iHS is known to be sensitive to ongoing or incomplete selection signatures, whereas XP-EHH is best at revealing the selection signatures close to fixation^44^. SHAPEIT2^45^ set to the default options (window 0.5, burn 200, prune 200, main 500) was used to obtain phased haplotypes for iHS and XP-EHH analyses, implemented using the rehh package^46^ in R. Candidate selection sweep regions were defined as the SNP regions under selection by both the applied statistics. Genes spanning ~ 100 kb upstream and downstream of the candidate selection regions were retrieved from the genome browser window of the Spud DB database (http://spuddb.uga.edu/).

## Supplementary Information

Supplementary Information 1.

Supplementary Information 2.

Supplementary Information 3.

Supplementary Information 4.

Supplementary Information 5.

Supplementary Information 6.

Supplementary Information 7.

Supplementary Information 8.

Supplementary Information 9.

Supplementary Information 10.

Supplementary Information 11.

Supplementary Information 12.

Supplementary Information 13.

Supplementary Information 14.
