# Supplementary material for: Analysis of genetic diversity and population structure among cultivated potato clones from Korea and global breeding programs
Source: Sci Rep. 2022 Jun 21;12:10462. doi: 10.1038/s41598-022-12874-2 (PMC9213424; doi:10.1038/s41598-022-12874-2)
Supplement: Supplementary file 1 — Supplementary Information 1. [file 41598_2022_12874_MOESM1_ESM.docx]

**Supplementary Information**

**Genetic characterization of global cultivated potato clones, including Korean potatoes, using genome-wide single nucleotide polymorphism markers**

Kwang Ryong Jo^1†^, Seungho Cho^2†^, Ji-Hong Cho^1^, Hyun-Jin Park^1^, Jang-Gyu Choi^1^, Young-Eun Park^1^, Kwang-Soo Cho^3^*

^1^Highland Agriculture Research Institute, National Institute of Crop Science, Rural Development Administration, Pyeongchang 25342, Republic of Korea

^2^Department of Central Area Crop Science, National Institute of Crop Science, Rural Development Administration, Suwon 16429, Republic of Korea

^3^Department of Southern Area Crop Science, National Institute of Crop Science, Rural Development Administration, Miryang 50424, Republic of Korea

*Corresponding author: kscholove@korea.go.kr

^†^These authors contributed equally to this work

**Table S1**; Germplasm information.

The sheet1 describes a 110-line Korean potato germplasm maintained at the Highland Agriculture Institute, NRC, RDA, Korea.

In the sheet2 is a total of 94 Japanese potatoes provided by Hosaka.

In the sheet3 is a total of 189 potato clones used in Schmitz Carley *et al* (2017). Descriptions of the clones were from Hirsch *et al* (2013).

The sheet5 gives information on sample clones used in revealing reproducibility of dosage genotyping calls methods.

**Table S2**; Dosage genotype calls generated by the GenomeStudio for a 110-line Korean potato germplasm. After filtering, 6575 SNP markers remained. Information on marker name, chromosome, chromosome position, SNP and maf (minor allele frequency) was given.

**Table S3**; Dosage genotype calls produced by the ClusterCall for a 393-line global potato genetic diversity panel. After filtering 3977 SNP markers remained. Information on marker name, chromosome, chromosome position, SNP and maf (minor allele frequency) was given.

**Table S4**; A tetraploid format STRUCTURE input file for a 110-line Korean potato germplasm

**Table S5**; Comparisons of reproducibility of the three different dosage genotype calling methods which have been developed based on different models. The individual sheets named GenomeStudio, ClusterCall and polyBreedR shows dosage genotype calls between replicates for each of samples which were generated using the GenomeStudio, ClusterCall and the function *geno_call* of polyBreedR, respectively. The sheet named the discordant_profile shows genotype calls with discrepancies between replicates for the three different dosage genotype calling methods.

**Table S6**; Population genetics summary based on diploid model using snpReady R package.

The sheet1 shows population genetics statistics computed by snpReady for Korean potato germplasm.

The sheet2 shows comparison of heterozygosity values based on diploid model and tetraploid model.

**Table S7**; The potato clones and quality controlled dosage genotype calls used in KLFDAPC analysis.

**Table S8**; HC input format using 3977 SNP markers for a 393-line genetic diversity panel.

**Table S9**; Inferred clusters by STRUCTURE, DAPC and HC using 6575 SNP markers for a 110-line Korean potato germplasm.

**Table S10**; The inferred cluster by DAPC using 3977 markers for a 393-line global genetic diversity panel.

The sheet1 shows the inferred clusters by DAPC using 3977 markers for a 393-line global genetic diversity panel.

The sheet2 shows the clustering of Korean potatoes inferred by DAPC for the 110-line panel and 393-line panel.

**Table S11**; The percent heterozygosity using 3977 SNP markers for a 393-line genetic diversity panel.

**Table S12**; The informativeness of 3977 SNP markers.

The informativeness was calculated based on the number of transitions across the samples for the genotype calls of each of 3977 SNPs.

**Table S13**; SNP loci under selection identified by iHS and XP-EHH approaches.

Clones used for selection signatures analysis in the sheet1.

SNP loci under selection identified by iHS and XP-EHH approaches are in the sheet2.

Also, .gff3 files retrieved from the Spud DB are available for genes ~100 kb upstream and downstream of the candidate selection regions.


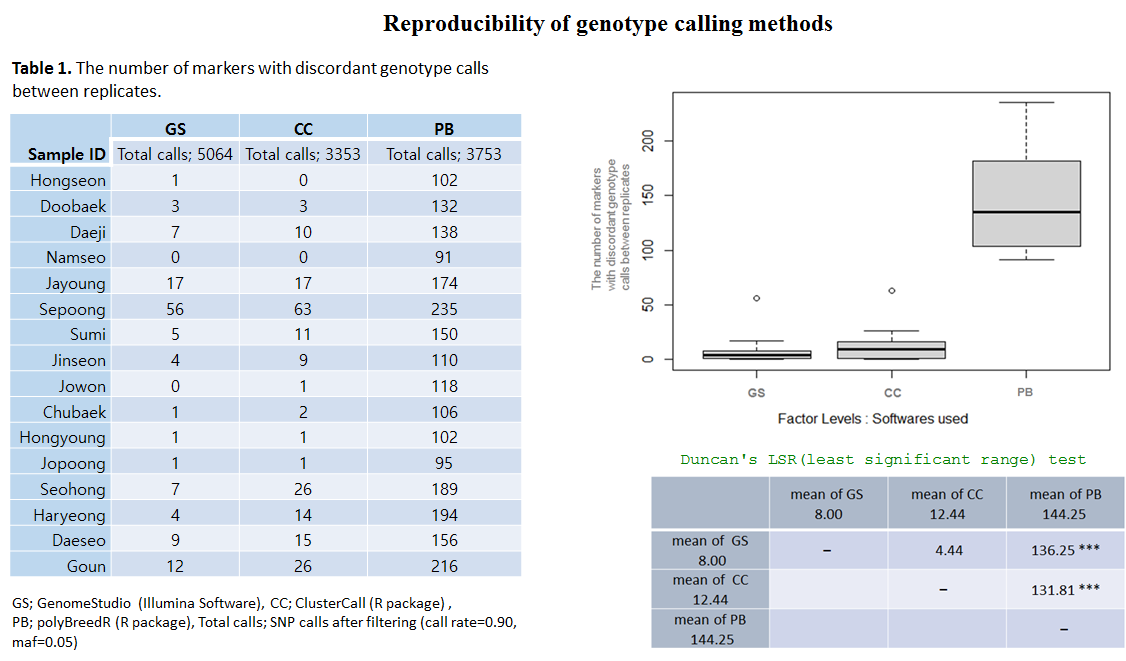


**Figure S1**. Reproducibility of dosage genotype calling methods. On the left side the Table 1 shows that the use of three different genotype calling softwares resulted in discrepancy in the number of markers with discordant genotype calls between three replicates for 16 samples. On the top right a box plot gives visual representations of the Table 1 and on the bottom right is shown the statistically significant discrepancy in the number of markers with discordant genotype calls.


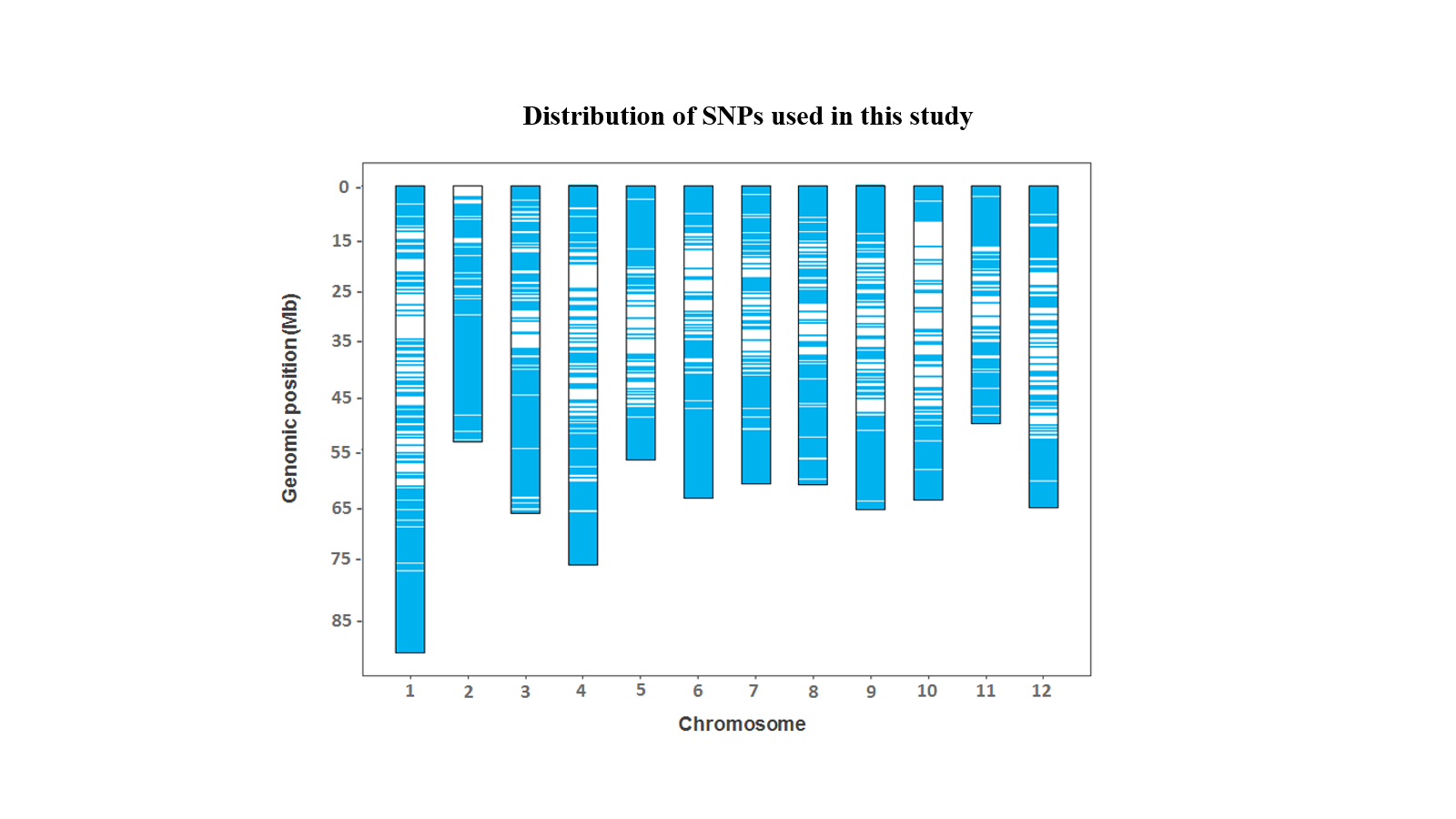


**Figure S2**. A diagram showing distribution of 6575 SNP markers across the twelve potato chromosomes. The blue bar indicates individual SNP markers. SNP density is shown to increase toward the ends of the chromosomes where gene density is higher.


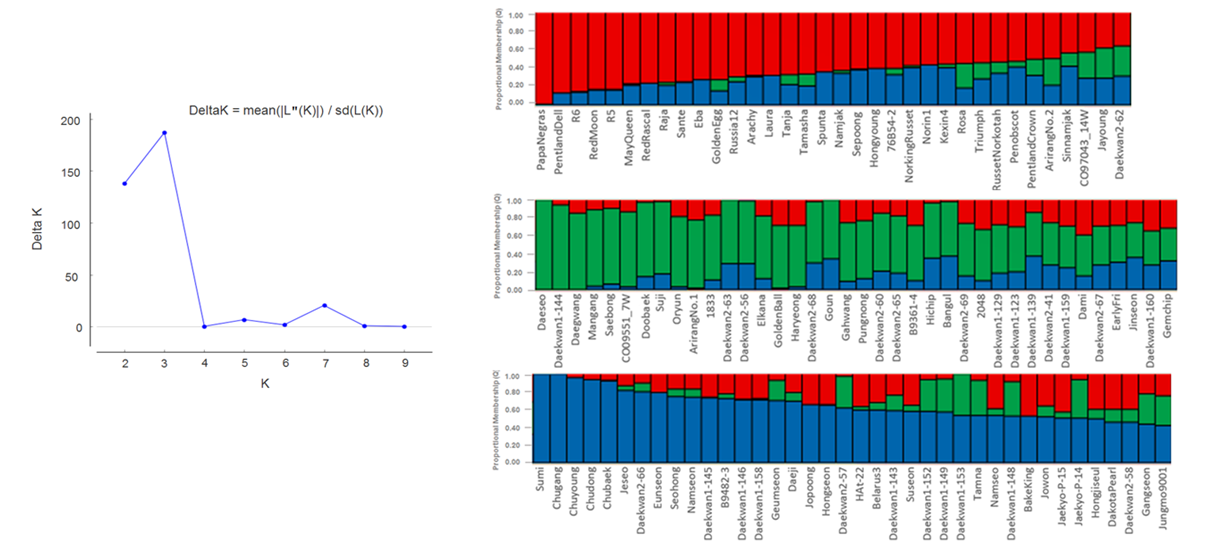


**Figure S3**. STRUCTURE analysis reveals three distinct subpopulations of the 110-line Korean germplasm panel based on 6575 SNP markers using the tetraploid model. The left shows estimation of the number of populations using LnP(D) derived ΔK. The maximum ΔK determined by structure harvester was K= 3. On the right is shown model-based clustering. The x-axis represents individual genotypes and the y-axis indicates the subpopulation membership.


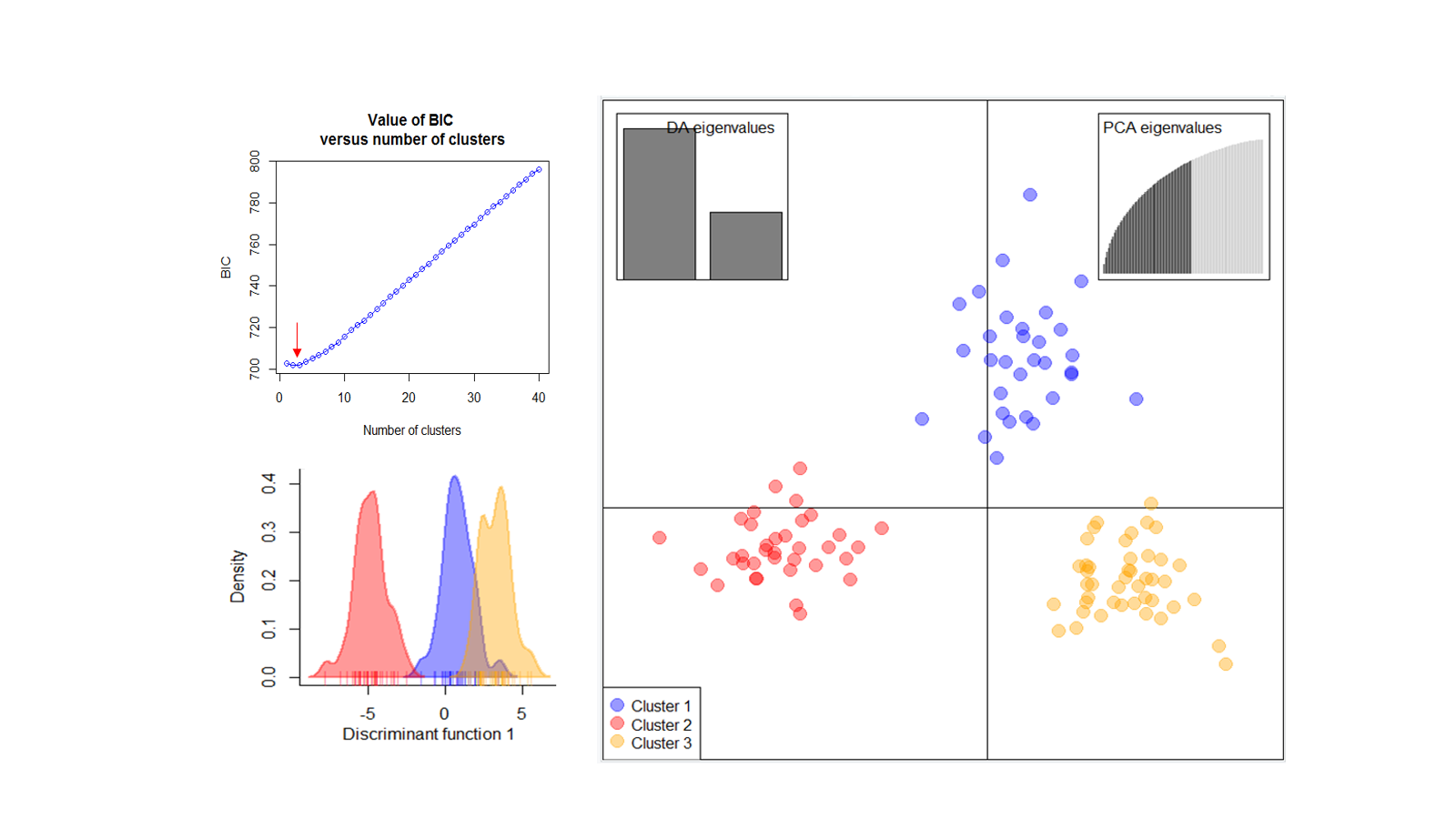


**Figure S4**. Discriminant analysis of principal components (DAPC) for 110 clones using 6575 SNP markers. The lowest Bayesian information criterion (BIC) value of K = 3 (top left) was obtained using the *find.clusters* function, confirming structured population. The density plot for the inferred clusters is on the bottom left. The axes represent the first two linear discriminants (LD) and small solid dots and ellipses represent each clone (right).


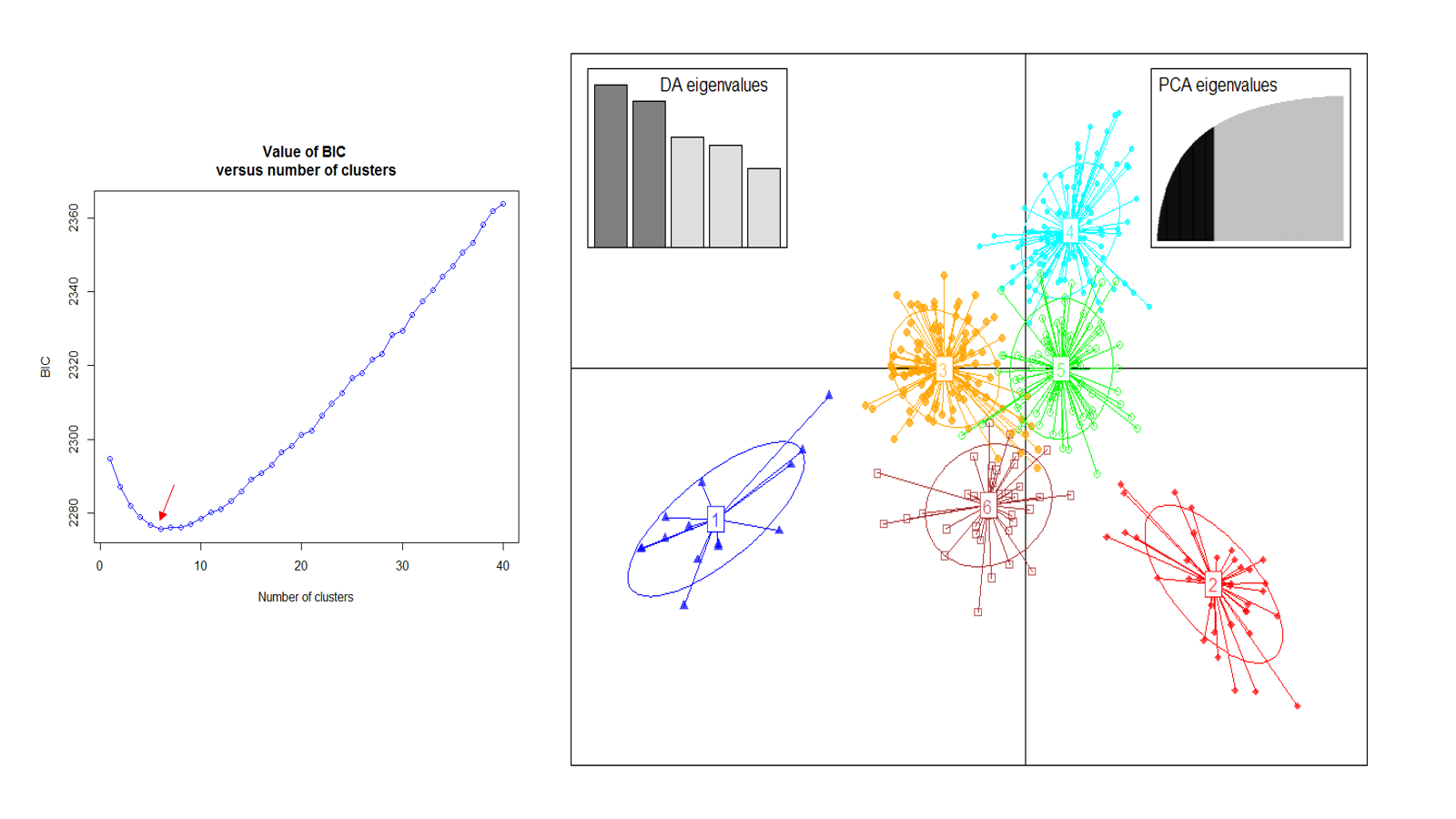


**Figure S5**. Discriminant analysis of principal components (DAPC) for 393 clones using 3977 SNP markers. The lowest Bayesian information criterion (BIC) value of K = 6 was obtained using the *find.clusters* function (left). The axes represent the first two linear discriminants (LD) and small solid dots and ellipses represent each clone (right). Numbers in circles indicate the different subpopulations identified by DAPC analysis.


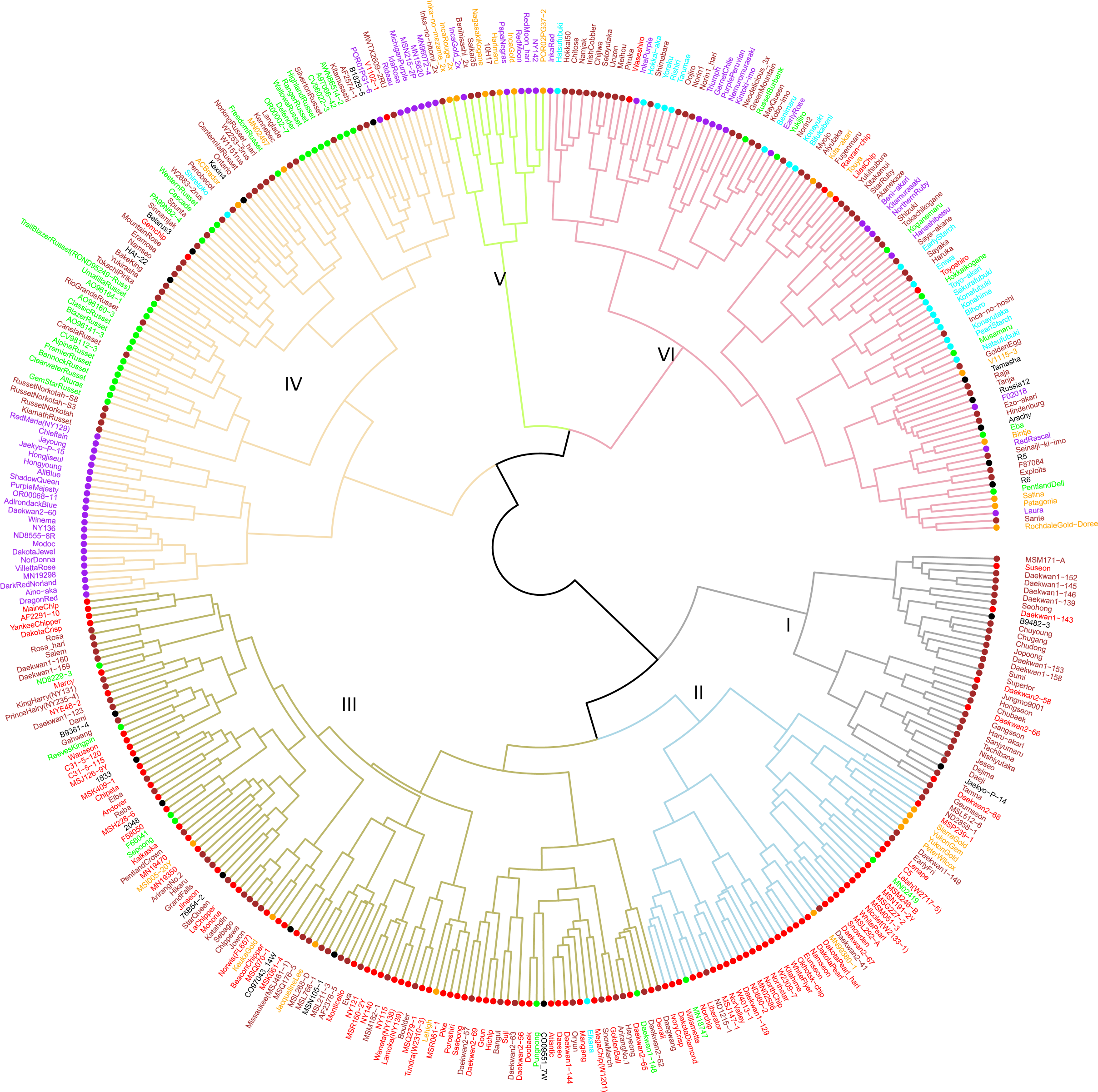


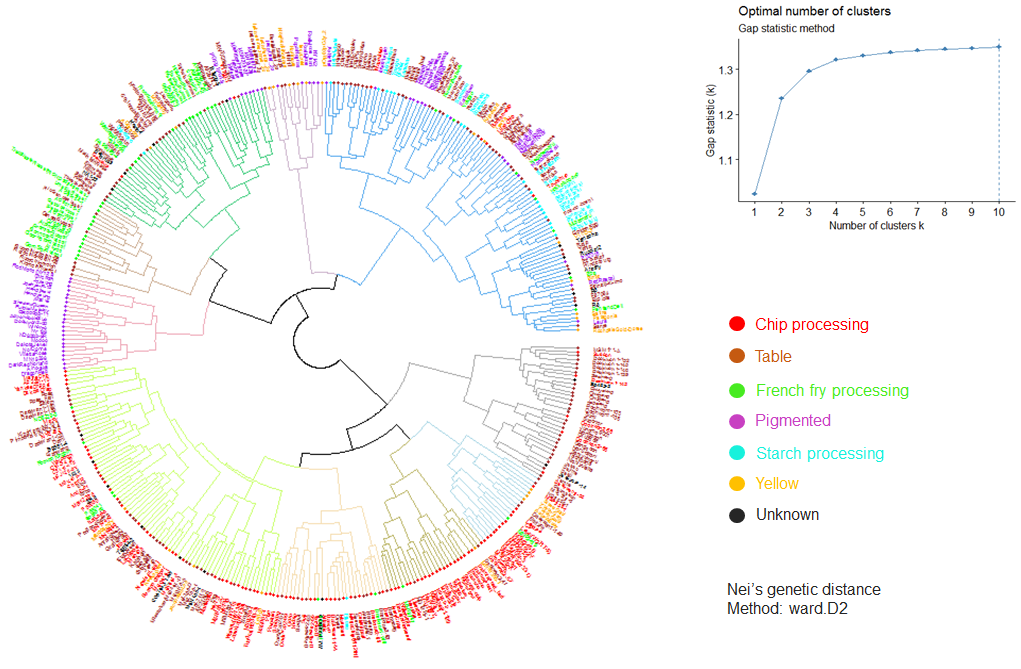


**Figure S6**. Hierarchical clustering (HC) for a global 393-line diversity panel using 3977 SNPs.

The dendrogram was constructed based on hierarchical clustering (Nei’s genetic distance, method = “ward.D2”) using 3977 SNP markers. We can observe two major clusters, each of which consist of three subgroups, i.e., I, II and III, and IV, V and VI, respectively. The color of leaves and clones represents the market class.


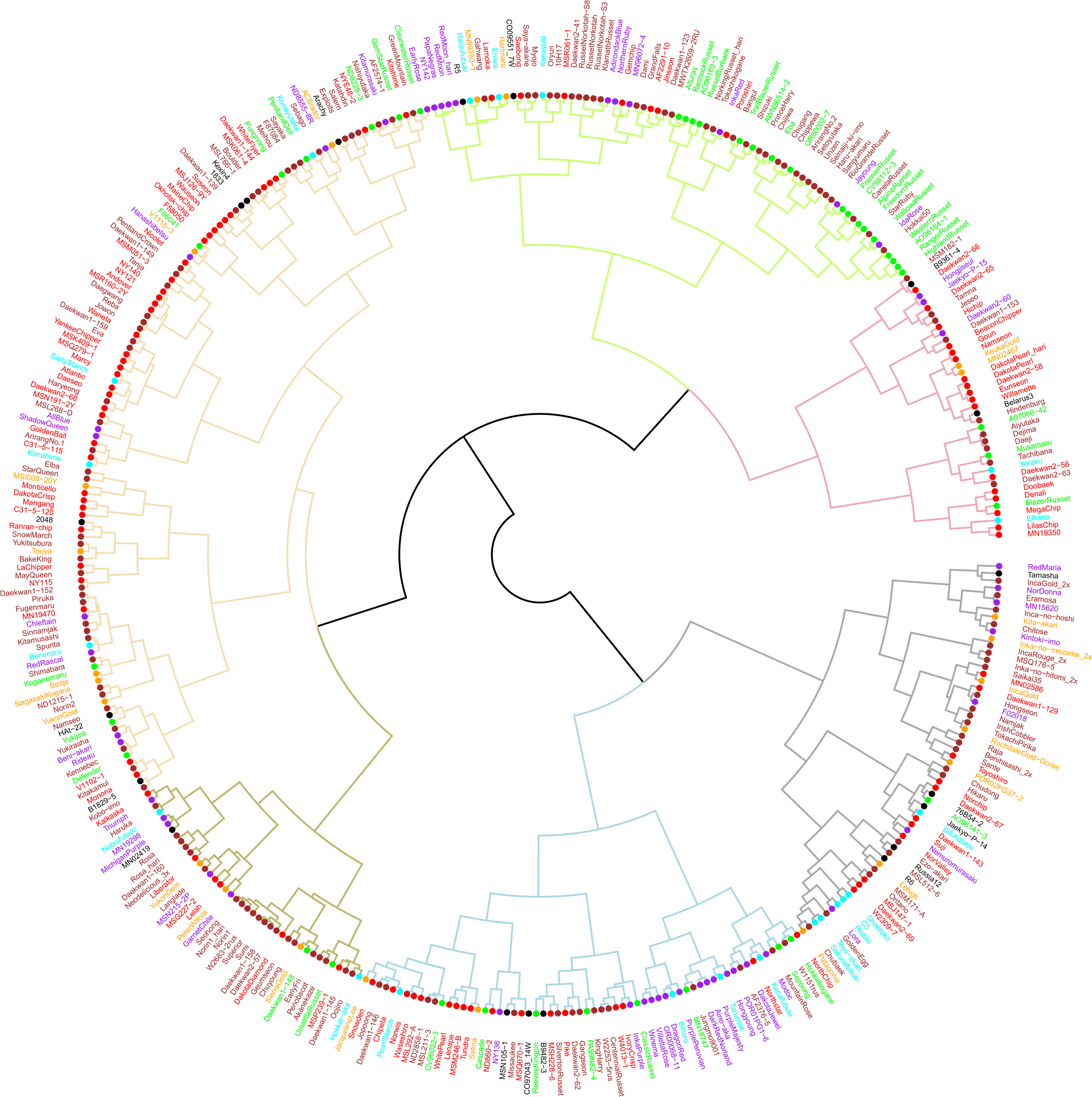


**Figure S7**. Using the selected 10 SNP-set to discriminate all clones in the global 393-line diversity panel. The dendrogram was constructed based on hierarchical clustering (Nei’s genetic distance, method = “ward.D2”) using ten highly informative SNP markers. Although the use of the 10 SNP-set enables discriminating all of 393 potato clones, please note that it does not have the phylogenetic ability corresponding to the use of 3977 markers. The color of leaves and clones represents the market class as described in Figure S6.
